# Sustained production and purification of *Ellipsomyxa mugilis* actinospores in a laboratory mesocosm

**DOI:** 10.1017/S0031182025100784

**Published:** 2025-09

**Authors:** Mónica Sá, Gabriel Oliveira, Carlos Antunes, Luís Filipe Rangel, Miguel Silva, Pedro Rodrigues, Sónia Rocha

**Affiliations:** 1 Institute for Research and Innovation in Health (i3S), University of Porto, Porto, Portugal; 2 School of Medicine and Biomedical Sciences (ICBAS), University of Porto, Porto, Portugal; 3 Interdisciplinary Centre of Marine and Environmental Research (CIIMAR), University of Porto, Matosinhos, Portugal; 4 Aquamuseu do Rio Minho, Vila Nova de Cerveira, Portugal

**Keywords:** *Chelon ramada*, fluorescence-activated cell sorting, *Hediste diversicolor*, life cycle, mesocosm, myxosporea, viability dyes, wheat-germ agglutinin

## Abstract

The lack of commercial treatments or vaccines against myxozoan parasites underscores the urgent need for a deeper understanding of the parasite infection in the fish and annelid hosts. Yet, progress in this research area is hindered by the lack of *in vitro* culture systems and the scarce number of *in vivo* models available. In addition, it is crucial to develop new protocols for the purification of spores and early developmental stages, as contamination of isolated parasite populations with host material remains a major obstacle for downstream biological applications, including the preparation of next-generation sequencing libraries, proteomics, pathology and immunological studies. Here, we lay the foundation for establishing the life cycle of *Ellipsomyxa mugilis* in an indoor laboratory mesocosm by co-housing thinlip grey mullet *Chelon ramada* and polychaetes *Hediste diversicolor*. A sustained infection of *H. diversicolor* was achieved, providing enduring access to *E. mugilis* actinospores. A purification protocol for actinospores was also developed using fluorescence-activated cell sorting and the lectin wheat-germ agglutinin in conjunction with 2 viability dyes, DAPI and propidium iodide, yielding a significantly pure parasite population with approximately 98% viability. This work establishes the basis for the development of a new myxozoan *in vivo* model and provides an effective, simple and rapid procedure for purifying viable *E. mugilis* actinospores. Together, these advances establish a framework for future studies on actinospore infectivity in the fish host.

## Graphical abstract

**Figure:**
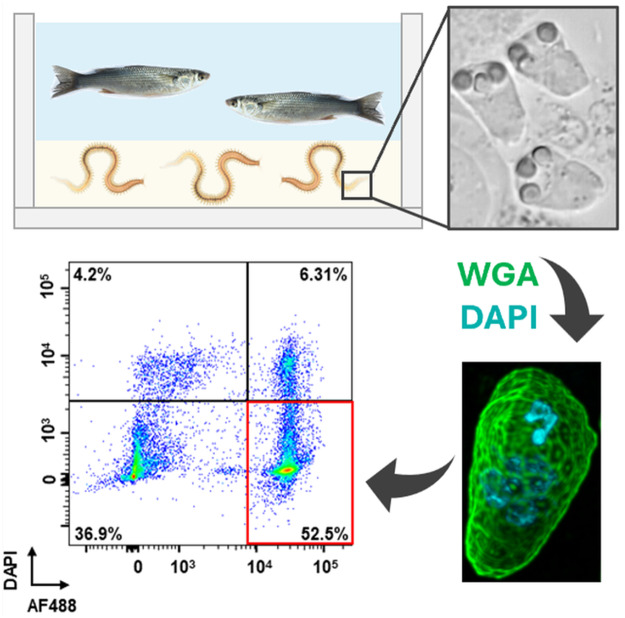

## Introduction

Myxozoans represent one of the most diverse and economically impactful groups of fish parasites worldwide (Shivam et al., 2021). These are cnidarian endoparasites with a complex life cycle that most commonly involve fish as intermediate hosts and annelid worms as definitive hosts. Transmission between hosts is achieved via the production of multicellular spores composed of external valve cells that surround infectious amoeboid cells (sporoplasms) and polar capsules containing polar tubules responsible for anchoring to host surfaces during invasion (Okamura et al., 2015). In the annelid host, the parasite multiplies asexually and undergoes gametogony before initiating sporogony to produce infectious actinospores within pansporocysts, located in the intestinal epithelium or coelomic cavity. Once released, actinospores drift passively in the water column and attach to fish host tissues, initiating infection through entry of the sporoplasm via the skin, gills or intestine. Following sporoplasm entry, presporogonic development occurs, during which the parasite multiplies and disseminates throughout the host body, eventually reaching the sporulation site – typically a specific tissue or organ cavity. There, the other infective stage – the myxospore – is produced and, when shed into the aquatic environment, settles in the water column to find a suitable annelid host, thereby perpetuating the cycle (Eszterbauer et al., 2015; Feist et al., 2015).

Despite their economic relevance for aquaculture industries, there are no effective prophylactic or therapeutic strategies available against myxozoans, and the number of *in vivo* models for studying host–parasite interactions at the cellular and molecular levels is very limited (Holzer et al., 2021). Of the approximately 2600 myxozoan species described so far (Lisnerová et al., 2022; Zhao et al., 2023), about 60 have their life cycle disclosed (Eszterbauer et al., 2015; Rangel et al., 2015, 2016, 2017; Rocha et al., 2019a, 2020), and of those, only 7 species have been maintained in the laboratory as experimental models, namely *Myxobolus cerebralis, Myxobolus pseudodispar, Ceratonova shasta, Enteromyxum leei, Enteromyxum scophthalmi, Tetracapsuloides bryosalmonae* and *Sphaerospora molnari* (Holzer and Holland, 2022). Moreover, with the advent of high-throughput sequencing (HTS), myxozoan omics studies have become critical for understanding host–parasite interactions and the host immune response towards these parasites. However, host contamination poses a significant challenge to the generation of good-quality myxozoan HST datasets, potentially leading to the misinterpretation of data and inaccurate conclusions regarding their biological significance (Alama-Bermejo and Holzer, 2021).

Several protocols have been tested to purify myxozoan spores from host cellular material, including density gradient centrifugation using sucrose (e.g. Andree et al., 1998, 1999; Gu et al., 2020; Guo et al., 2020; Xiao et al., 2022) and Percoll^TM^ (e.g. Andree et al., 1998, 1999; Chase et al., 2001; Knaus and El-Matbouli, 2005b; Whipps and Kent, 2006; Kaltner et al., 2007; Yokoyama et al., 2014; Piriatinskiy et al., 2017; Brekhman et al., 2021; Xiao et al., 2022), and a dextran-polyethylene glycol 2-phase system (Holzer et al., 2003; Eszterbauer and Székely, 2004). More recently, Born-Torrijos and colleagues developed a protocol for purification of *S. molnari* proliferative stages from fish blood using diethylaminoethyl cellulose (DEAE-C) ion exchange chromatography, achieving high parasite purity, survival and infectivity (Born-Torrijos et al., 2022). Finally, fluorescence-activated cell sorting (FACS), a high-throughput technique considered the gold standard for cell separation (Sutermaster and Darling, 2019), was recently applied to purify *T. bryosalmonae* from infected kidney tissue, resulting in a higher enrichment of parasite reads in the RNA-seq data compared to previous studies (Shivam et al., 2023). FACS is a highly selective technique, in which a specific cell population can be labelled – typically with a fluorescent dye or antibody – and isolated based on its fluorescent signal and other morphological parameters, such as size and granulometry (Dia and Cheeseman, 2021).

Nonetheless, new purification protocols are needed for myxozoans to increase the parasite-to-host-cell ratio, including for actinospores and early developmental stages (Alama-Bermejo and Holzer, 2021). Here, we have established an indoor mesocosm system that houses both hosts of *Ellipsomyxa mugilis* – *Hediste diversicolor* polychaetes and thinlip grey mullet *Chelon ramada* (Sitjà Bobadilla and Alvarez‐Pellitero, 1993; Rangel et al., 2009) – and achieved a sustained production of actinospores. Moreover, we report the first FACS-based protocol for the purification of viable, i.e. living and potentially infectious, myxozoan actinospores. By combining the use of a fluorescent wheat-germ agglutinin (WGA) conjugate – a lectin shown to bind to myxospores from multiple species, including *E. mugilis* (Lukeš et al., 1993; Muñoz et al., 1999) – with 4′,6-diamidino-2-phenylindole (DAPI) and propidium iodide (PI), we developed a sorting procedure that enables the purification of surface-stained actinospores from host cells, achieving high purity and viability rates in the sorted population.

## Material and methods

### Fish and polychaetes

Forty-five specimens of thinlip grey mullet *C. ramada* about 10 cm long and 100 adult *H. diversicolor* polychaetes were obtained from Portuguese estuaries (Minho River and Aveiro estuary, respectively), where *E. mugilis* is endemic. They were transported to the School of Medicine and Biomedical Sciences (ICBAS) animal facility and placed in a 0.46 m^3^ closed recirculation tank with aeration. To ensure easy access, polychaetes were kept in a smaller compartment within the main tank, which contained a 10 cm layer of sand to support their development. Sand was previously sterilized to render it azoic. The sampling compartment was covered with a mesh to allow free water circulation while preventing direct contact between the mullet and the polychaetes. The system was maintained with artificial saltwater kept at 18 ± 2 °C and with salinity between 17 and 20‰, under a 12-h light/dark photoperiod. Fish and polychaetes were fed daily with commercial pellet diet (Tetra). The system was monitored daily and, once a week, the levels of ammonia and nitrates were measured using ammonium/ammonia and nitrite-test kits (Sera). Partial water renewal was performed to ensure optimal water quality with ammonia and nitrite levels maintained below 0.25 and 0.05 mg L^−1^, respectively. To prevent excessive life cycle shortening and adult size decrease of *H. diversicolor* over time, small polychaetes were removed, and approximately 20 wild adult individuals were added to the system when only very small mature polychaetes could be collected.

### Analysis of fish and polychaete infection status by microscopy and PCR

Sampling was conducted after 6 months of cohabitation to (i) cover the acclimation and quarantine period of fish and polychaetes and (ii) align with the expected developmental time of *E. mugilis* in polychaetes (∼28 days; Rangel et al., 2009) and related species in fish (3–4 months; Køie et al., 2004). Hence, after a 6-month cohabitation period, a random sample of approximately 10% of the *C. ramada* population (*n* = 4) was examined for the presence of *E. mugilis* plasmodia and mature myxospores in the bile and gallbladder using microscopy. These tissues were also preserved in 100% ethanol and stored at 4 °C for subsequent molecular screening. Similarly, the presence of *E. mugilis* actinospores and other parasite developmental stages in the coelom of polychaetes was assessed by microscopy. Briefly, 20 polychaetes were collected from the tank, and their posterior segments were sectioned with a scalpel blade and gently pressed with a coverslip to release the coelomic fluid. When infected, polychaetes were kept individually and monitored for actinospore development following the procedures described by Rangel et al. (2009). Polychaetes lacking microscopic signs of infection were returned to the tank, but their posterior segments were stored in 100% ethanol at 4 °C for molecular screening.

Polychaetes and fish genomic DNA (gDNA) were extracted using a previously described phenol-chloroform-based method (Holzer et al., 2004). In brief, pellets were re-suspended in TNES buffer [10 mM Tris–HCL (pH 8), 125 mM NaCl, 10 mM EDTA, 0.5% SDS, 4 M urea] and digested overnight at 55 °C with 0.1 mg mL^−1^ proteinase K (Merck). Following the addition of phenol-chloroform to the cell lysate, nucleic acids were precipitated from the aqueous phase with ethanol and eluted in nuclease-free water. Samples were stored at −20 °C until further use.

Polymerase Chain Reactions (PCRs) were performed using Supreme NZYTaq II DNA polymerase (NZYtech) according to the manufacturer’s instructions. Reactions were set up using 10 pmol of each primer, 10 nmol of dNTPs, 2.0–2.5 mM MgCl_2_ and 1.25 units Taq DNA polymerase. The sequences of primers used in this study are shown in Table 1. To exclude the possibility of having false negatives in the *E. mugilis* molecular screening, the amount of gDNA used in the PCR reactions was optimized through the amplification of host control genes, namely *C. ramada* cytochrome c oxidase subunit 1 gene and mitochondrial serine, leucine and alanine transfer RNA genes of *H. diversicolor.* Fish gDNA was amplified using primer pair FishF1/FishR1 (Ward et al., 2005), with initial denaturation at 95 °C for 3 min, 35 cycles of 94 °C for 30 s, 52 °C for 30 s and 72 °C for 30 s, and final extension at 72 °C for 5 min. The conditions for amplifying *H. diversicolor* tRNA genes with the primer pair Hediste tRNA_F1/Hediste tRNA_R1 were similar to those described earlier, with the exception that primer annealing was performed at 47 °C, and the extension step in the cycles was shortened to 15 s.

**Table 1.** Oligonucleotides used in this study

Primer name	Sequence 5ʹ→3ʹ	Reference
FishF1	TCAACCAACCACAAAGACATTGGCAC	Ward et al. (2005)
FishR1	TAGACTTCTGGGTGGCCAAAGAATCA	Ward et al. (2005)
Hediste tRNA_F1	GGCAGAGTTATGCACTTGAC	This study
Hediste tRNA_R1	ACCTGGTTGATCCTGCCAG	This study
ERIB1	ACCTGGTTGATCCTGCCAG	Barta et al. (1997)
ERIB10	CTTCCGCAGGTTCACCTACGG	Barta et al. (1997)
Emugilis_F1	CGTCCGCTCGATCGTTTAAACC	This study
Emugilis_R1	GAGACTTGCGTCTCTGATGGC	This study

A nested-PCR strategy was used to screen for *E. mugilis* 18S rRNA gene in the annelids and fish tissues. The first round of amplification was carried out using universal eukaryotic primers ERIB1 and ERIB10 (Barta et al., 1997) and the following conditions: initial denaturation at 95 °C for 3 min, followed by 35 cycles of 95 °C for 1 min, 48 °C for 1 min and 72 °C for 1 min 45 s. A final extension step was carried out at 72 °C for 10 min. The second round of amplification was performed using primers Emugilis_F1/Emugilis_R1 with initial denaturation at 95 °C for 3 min, followed by 35 cycles of 94 °C for 30 s, 60 °C for 30 s and 72 °C for 15 s, and a final extension at 72 °C for 5 min. gDNA of non-purified *E. mugilis* actinospores was used as positive control. PCR products were sequenced by Sanger sequencing (STAB VIDA, Portugal) and verified against the *E. mugilis* 18S rRNA partial sequence with accession number MK193812.1 available in the NCBI GenBank database (https://www.ncbi.nlm.nih.gov/genbank/, accessed on 14 June 2023), using the Basic Local Alignment Search Tool.

### Collection of actinospores

To collect actinospores, polychaetes were placed on top of a cavity microscope slide, and their posterior end was gently separated from the rest of the body using a pair of dissecting needles attached to 1 mL syringes. The separated segments were flushed with Dulbecco’s Phosphate Buffered Saline (DPBS; Biowest), and the drained fluid containing the parasites was collected into a clean microcentrifuge tube placed on ice. Actinospore suspensions were filtered through a 40-µm cell strainer (pluriStrainer), centrifuged at 1500 g for 3 min and washed with fresh DPBS. The total number of actinospores collected and their viability was determined using a Neubauer counting chamber and the trypan blue exclusion assay.

### Immunofluorescence assays

WGA specificity to actinospores was analysed by widefield fluorescent microscopy. Parasites were collected as described earlier, labelled with 5 µg mL^−1^ Alexa Fluor 488-conjugated WGA (WGA-AF488; Invitrogen™) for 30 min at room temperature and washed with DPBS. Slides were immediately imaged using an upright epifluorescence Eclipse E400 microscope (Nikon) and a Nikon DS-Fi3 digital camera (Nikon). Images were processed using ImageJ/Fiji software (ImageJ, National Institutes of Health). To analyse the WGA-binding pattern by confocal microscopy, microscopic slides (Marienfeld Superior) were coated with poly-L-lysine (Sigma), following the manufacturer’s recommendations. Collected actinospores were then seeded onto the coated slides and fixed with paraformaldehyde 4% (w/v) in DPBS for 30 min at room temperature. Following a washing step with DPBS, actinospores were labelled with 5 µg mL^−1^ WGA-AF488 for 30 min at room temperature and washed again. Slides were mounted in VectaShield^®^ mounting medium containing DAPI (Vector Laboratories) and imaged using an inverted epifluorescence Leica DMI6000 microscope (Leica Microsystems) and LAS X software version 3.7.4.23463 (Leica Microsystems). Confocal microscopy was conducted using Leica Scanning Confocal SP8 (Leica Microsystems) and LAS X software. Images were deconvolved using Huygens software (Scientific Volume Imaging) and processed using ImageJ/Fiji software.

### Flow cytometry assays

To evaluate the use of WGA for actinospore labelling and subsequent isolation by FACS, actinospores were labelled with 5 µg mL^−1^ WGA-AF488 for 30 min at room temperature, washed, re-suspended in fresh DPBS and analysed by flow cytometry. Actinospores incubated only with DPBS were used as control. To identify WGA-AF488-stained actinospores, parasites were first gated based on their forward and side scatter characteristics. Then, cell doublets were excluded, and finally, WGA-stained actinospores were identified based on the AF488 signal intensity.

To sort only viable actinospores and to assess the viability of the sorted parasite population, several membrane-impermeant dyes were evaluated as viability markers for our FACS protocol, as follows. Actinospores were subjected to heat stress at 60 °C for 10 min to induce parasite cell death. The percentage of non-viable actinospores was then assessed using a standard trypan blue exclusion assay and compared to the percentage of DAPI-, PI- and 7-aminoactinomycin D (7-AAD)-positive cells quantified by flow cytometry. Thus, prior to acquisition, actinospores were either re-suspended in (i) DPBS 0.01 μg mL^-1^ DAPI (Merck); (ii) DPBS 5 µg mL^-1^ PI (Thermofisher Scientific) for 15 min on ice, washed and re-suspended in fresh DPBS; or (iii) DPBS 0.83 µg mL 7-AAD (BioLegends). Actinospores incubated only with DPBS, were used as controls.

For sorting experiments, actinospores were labelled with 5 µg mL^−1^ WGA-AF488 for 30 min at room temperature, washed and re-suspended in DPBS 0.01 µg mL^−1^ DAPI. AF488^+^/DAPI^−^ actinospores were first gated based on their forward and side scatter characteristics. After the exclusion of cell doublets, viable actinospores were identified as AF488^+^/DAPI^−^ events and sorted from the remaining host cells. Post-sorting analyses were performed to assess the purity – defined as the percentage of the sorted events that were AF488^+^/DAPI^−^ – and the viability of the sorted population. In turn, viability was assessed following staining with 5 µg mL^−1^ PI: after initial gating the actinospores based on their AF488 and DAPI fluorescence intensity (AF488^+^/DAPI^−^), the percentage of PI^−^ events (AF488^+^/DAPI^−^/PI^−^) was calculated. Purity and viability values were obtained from 3 independent experiments using actinospores collected from different polychaetes. Unstained actinospores and actinospores labelled only with WGA-AF488, DAPI or PI were used as controls. To assess WGA specificity to actinospores, coelomic cells collected from an uninfected polychaete, were processed and stained with WGA-AF488 and DAPI as described earlier, and were used as an additional control.

Flow cytometry experiments were performed at 4 °C using a BD FACSAria™ II and BD FACSAria™ Fusion (BD Biosciences). The analysis of flow cytometry data was performed using FlowJo v10.10.0. Following sorting, actinospore suspensions were observed under a light microscope to check for the presence of host cells and debris.

## Results

### *Sustained production of* Ellipsomyxa mugilis *actinospores in polychaetes under laboratory conditions*

**Figure 1.**
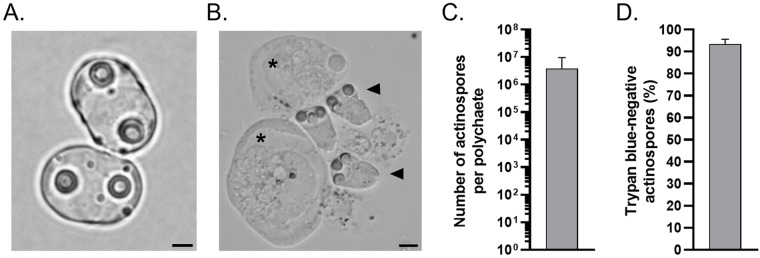
*Ellipsomyxa mugilis* infection in *Chelon ramada* and *Hediste diversicolor*. (A, B) Light micrograph of *E. mugilis* myxospores (panel A) and actinospores (panel B; arrowheads) collected from an infected mullet and polychaete, respectively. Host cells are highlighted by asterisks. Scale bars: 2.5 µm (panel A) and 5 µm (panel B). (C) Number of actinospores collected from infected polychaetes (*n* *=* 16). Bars indicate the mean + SD. (D) Viability of actinospores assessed using the trypan blue exclusion assay, represented as the percentage of trypan blue-negative actinospores. Bars indicate the mean + SD of independent experiments using actinospores collected from different polychaetes (*n* *=* 5).

After a 6-month period of cohabitation between wild *C. ramada* and *H. diversicolor* polychaetes obtained from *E. mugilis* endemic areas, microscopic evidence of *E. mugilis* developmental stages was observed in 25% of fish samples (Figure 1A, *n* = 4), whereas 75% tested positive for this parasite DNA in the gall bladder. Similarly, molecular screening of annelids showed that 85% of individuals (*n* = 20) were positive for parasite DNA, while only 24% exhibited *E. mugilis* development in their coelomic cavity. Although PCR-positive annelids included females, males and individuals of undetermined sex, only the latter 2 developed infections that resulted in the production of mature actinospores (Figure 1B).

High numbers of actinospores were consistently recovered from polychaetes, ranging from approximately 3.63 × 10^5^ to 2.45 × 10^7^ per infected individual (Figure 1C), with 93.6 ± 2.1% viability (Figure 1D). The infection was sustained over time across multiple generations of polychaetes, as about 30% of juvenile specimens that emerged in the tank successfully developed infection.

### *Wheat-germ agglutinin binds to the surface of* Ellipsomyxa mugilis *actinospores*

Actinospores collected from polychaetes were frequently found alongside a substantial amount of host cells and debris (Figure 1B), compromising their direct use in omics experiments. To overcome this challenge, actinospores were labelled with WGA, a lectin capable to bind to the valves of *E. mugilis* myxospores (Muñoz et al., 1999), as part of a flow-cytometry–based strategy.

**Figure 2.**
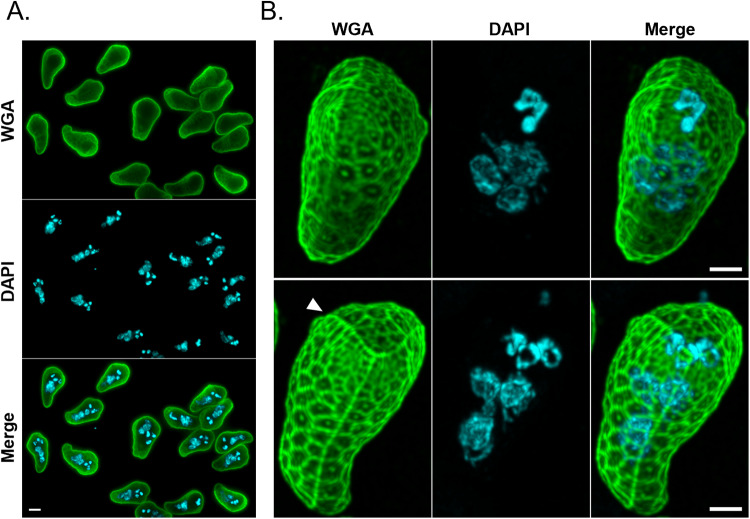
WGA binds to the surface of *Ellipsomyxa mugilis* actinospores. Representative immunofluorescence images of *E. mugilis* actinospores stained with Alexa Fluor 488-conjugated WGA (green) and DAPI (cyan), acquired using widefield (panel A) and confocal microscopy (panel B). Panel B corresponds to maximum Z-projections of 95 confocal images separated by 0.25 µm. Arrowhead indicates the suture line. Scale bars: 5 µm (panel A) and 2.5 µm (panel B). WGA, wheat-germ agglutinin; DAPI, 4′,6-diamidino-2-phenylindole.

As shown in Figure 2A, a strong fluorescent signal was detected at the surface of non-permeabilized parasites, suggesting that WGA interacts either with *N*‐acetylglucosamine or *N*‐acetylneuraminic acid residues present in the valve cells of *E. mugilis* actinospores. The WGA-labelling pattern resembled a honeycomb-like framework covering the actinospore shell valves and suture lines, with a distinct dot-like fluorescent spot appearing at the centre of each alveolus (Figure 2B). Importantly, WGA binding was specific to actinospores, exhibiting minimal to no binding to host cells (Supplementary Figure S1).

### Purification of viable actinospores by fluorescence-activated cell sorting

**Figure 3.**
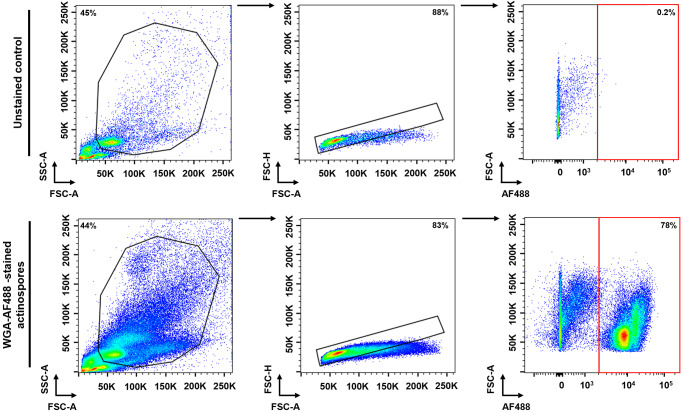
Detection of WGA-stained actinospores by flow cytometry. Flow cytometry dot plots showing the gating strategy used to identify actinospores stained with Alexa Fluor 488-conjugated WGA (WGA-AF488). From left to right: actinospores were first gated based on the forward and side scatter properties, then gated for single parasites and identified based on the AF488-fluorescence intensity. As negative control, actinospores incubated with DPBS were used (upper panel). WGA, wheat-germ agglutinin; DPBS, Dulbecco’s Phosphate Buffered Saline.

When stained with the fluorescent WGA conjugate, actinospores were distinguishable from host cells by an increase in the AF488 signal intensity (Figure 3). Regarding the effectiveness of DAPI, PI and 7-AAD to discriminate dead from live actinospores, our viability assay revealed percentages of DAPI- and PI-positive actinospores similar to those obtained in the trypan blue exclusion assay, confirming their reliability in detecting non-viable actinospores. In contrast, 7-AAD failed to stain non-viable actinospores (Supplementary Figure S2). As a result, DAPI and PI were selected as viability dyes for subsequent sorting experiments.

**Figure 4.**
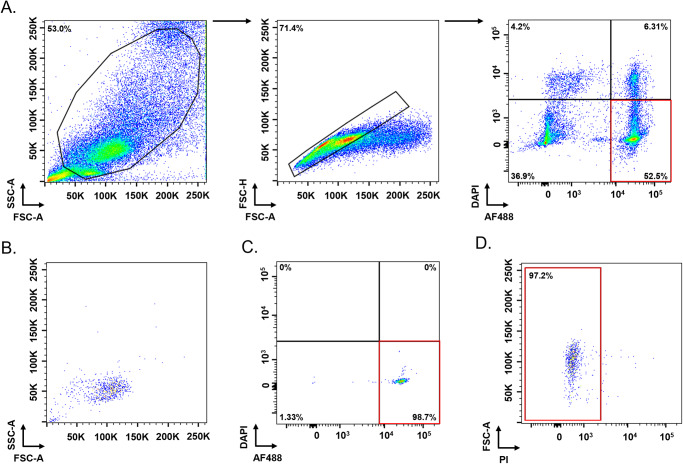
Purification of viable *Ellipsomyxa mugilis* actinospores by flow activated cell sorting. (A) Flow cytometry gating approach used to sort viable *E. mugilis* actinospores. From left to right: actinospores were first gated based on the forward and side scatter properties, then gated for single parasites and sorted using a AF488^+^/DAPI^−^ gate. (B–D) Representative dot plots of the post-sorting analyses. Panel B corresponds to ungated FSC vs SSC dot plot of sorted cells. Purity (panel C) and viability (panel D) of sorted cells, represented as the percentage of AF488^+^/DAPI^−^ and AF488^+^/DAPI^−^/PI^−^ events, respectively. DAPI, 4′,6-diamidino-2-phenylindole; FSC, forward scatter; SSC, side scatter.

Viable actinospores, represented as WGA^+^/DAPI^−^ events, were purified from host material using the gating strategy shown in Figure 4. Applying the same sorting strategy to coelomic cells from an uninfected polychaete revealed that only approximately 7% of events were WGA^+^/DAPI^−^ (Supplementary Figure S3), indicating that 93% of the host cells collected along with actinospores were not sorted with them. Importantly, WGA^+^/DAPI^−^ host cells exhibited fluorescence intensities approximately 32 times lower than those of actinospores (Supplementary Figure S4), minimizing their impact during the sorting experiments, as the threshold used to isolate WGA^+^/DAPI^−^ actinospores excluded low-intensity events.

Post-sorting analyses revealed high purity and viability of the sorted population, reaching 97.9 ± 1.1% and 97.7 ± 2.1%, respectively (Figure 4B–D). Together, these results validate the use of this method for the purification of *E. mugilis* actinospores.

## Discussion

### *Towards the establishment of an* Ellipsomyxa mugilis *mesocosm* model

The proliferation of myxozoan parasites and disease severity in fish correlates proportionally with water temperature, making these parasites an emerging threat to wild and farmed fish populations under global climate change (Okon et al., 2024). Renewed efforts should focus on developing new experimental models to study early stages of infection, particularly the infective actinospore and the subsequent proliferative stages, as they are promising candidates to search for vaccine targets (Holzer et al., 2021). Here, we laid the foundation for establishing the life cycle of *E. mugilis* in an indoor mesocosm system by co-housing thinlip grey mullet *C. ramada* and *H. diversicolor* polychaetes obtained from *E. mugilis* endemic areas.

Several criteria were considered when selecting *E. mugilis* as the myxozoan species to be used in the system. First, we chose a coelozoic species so that myxospores could be continuously released into the water through stool or urine, i.e. without the need to euthanize infected fish to ensure parasite transmission to the annelid host. *Ellipsomyxa mugilis*, originally described as *Zschokkella mugilis* (Sitjà Bobadilla and Alvarez‐Pellitero, 1993), is a coelozoic species that inhabits the gallbladder lumen of mugilids across the Mediterranean Sea, namely *C. ramada, Chelon saliens* and *Mugil cephalus* (Sitjà Bobadilla and Alvarez‐Pellitero, 1993; Thabet et al., 2016). In Portugal, infections by *E. mugilis* were reported in *C. ramada* from the estuary of the River Minho, 275 km north to the Aveiro estuary where *E. mugilis* actinospores were first observed in *H. diversicolor* polychaetes (Rangel et al., 2009; Rocha et al., 2019b). In these estuaries, infection prevalences in *C. ramada* and *H. diversicolor* were reported as 22.7% and 0.5%, respectively (Rangel et al., 2009; Rocha et al., 2019b). This knowledge allowed access to naturally infected wild populations of the fish and annelid hosts of *E. mugilis*. The size of the annelid host and the capacity to keep it in a co-housing system with *C. ramada* was also considered to ensure easy handling and a facilitated access to large numbers of actinospores.

After 6 months of co-habitation, 75% of the sampled fish tested positive for the presence of *E. mugilis* DNA in their gallbladder, although microscopic evidence of parasites was found in only 25% of the fish. This discrepancy in prevalence of infection may be explained by the higher sensitivity of PCR-based methods in comparison to more traditional approaches, as previously shown by Alama-Bermejo et al. (2023). In agreement, molecular screening of annelids consistently revealed a higher percentage of PCR-positive individuals compared to the percentage of infected polychaetes determined through microscopic examination of their coelomic fluid. Importantly, while molecular methods may detect early *E. mugilis* in the intestinal epithelium of polychaetes or even the earliest developmental stages in the coelomic fluid, they may also overestimate the prevalence of infection in the annelid population. Polychaetes with ingested myxospores or parasite environmental DNA may test positive in the molecular screening, even if they are unable to develop a successful infection. This may explain why some female individuals were PCR-positive in our study, despite actinospores being observed only in males and individuals of undetermined sex. These findings are congruent with the observations previously reported by Rangel et al. (2009), as natural infections of *E. mugilis* were found in male and individuals of undetermined sex, but not in females. Nevertheless, an infection prevalence of 24% was observed in polychaetes collected from the mesocosm, a value 48 times higher than that recorded in wild polychaetes (Rangel et al., 2009), demonstrating that *E. mugilis* transmission between fish and annelids occurred successfully within the system.

The transmission dynamics of *E. mugilis* in the mesocosm are not yet fully understood. Controlled experiments using naïve hosts are required to clarify the complex infection processes of *E. mugilis* in both fish and annelids and to establish a fully controlled life cycle in the laboratory. It also remains unclear whether infective myxospores are continuously discharged by fish or released only during a limited period of time. Because a high prevalence of infection was detected across multiple generations of polychaetes, the most likely scenario is that individuals were infected *de novo* through the ingestion of myxospores present in the environment. While vertical transmission was previously reported in other myxozoan species (Morris and Adams, 2006; Atkinson and Bartholomew, 2009), its contribution to the perpetuation of *E. mugilis* life cycle is unlikely. Some early studies report the occurrence of asexual reproduction by parthenogenesis in *H. diversicolor* populations; however, it has since been acknowledged that these observations resulted from the existence of a strong female-biased sex ratio in this species (Dales and Müller, 1950). Although productive *E. mugilis* infections have not been documented in females, future studies are required to understand whether vertical transmission might occur via eggs.

In our system, we were able to collect approximately 3.63 × 10^5^–2.45 × 10^7^ actinospores per polychaete, depending mostly on the size of the individual; that is, the larger the polychaete, the more actinospores could be collected. Under constant warm temperature and non-limiting food conditions, an increase in the somatic growth rate along with a decrease in the average body size of polychaetes was observed (data not shown), resulting in a shortened life cycle, as reported for other Nereididae (Olive, 1999). To mitigate this phenomenon, the polychaete population in the tank was closely monitored for individual number and size, with small individuals being removed and wild adult individuals being added as needed. Notwithstanding, we were able to routinely collect a large number of actinospores, which is crucial for future genomic studies, given the low gDNA yield obtained after extraction from actinospores. This low yield likely reflects both the reduced size of myxozoan genomes (Alama-Bermejo and Holzer, 2021) and the relatively low number of nuclei present in *E. mugilis* mature actinospores (Rangel et al., 2012). Finally, although our experiments show that most actinospores are viable following isolation according to our protocol, future studies are needed to determine whether the collected actinospores remain infective to the fish host.

### *The binding of WGA to* Ellipsomyxa mugilis *actinospores and possible biological implications*

Flushing the coelomic cavity of infected polychaetes led to the co-isolation of a considerable amount of host cells alongside actinospores. However, a parasite suspension free of host cells and debris is essential for innumerous biological assays, including, for example, the preparation of next-generation sequencing libraries, proteomics, pathology and immunological studies. Among the purification protocols available for myxozoan spores, FACS stands out due to its speed, sensitivity and high-throughput nature. Since the genome of *E. mugilis* has yet to be sequenced and no commercial anti-*E. mugilis* antibody is available, as in the case of *T. bryosalmonae* (Shivam et al., 2023), we have developed a flow cytometry-based purification protocol for actinospores using a fluorescent lectin conjugate.

Lectins, proteins containing a non-catalytic domain that bind to specific carbohydrates (Chettri et al., 2021), have long been used to map the carbohydrate content of myxozoan spores and other developmental stages (Castagnaro et al., 1991; Lukeš et al., 1993; Marin De Mateo et al., 1997; Muñoz et al., 1999, 2000; Morris and Adams, 2004; Knaus and El-Matbouli, 2005a; Kaltner et al., 2007; Redondo et al., 2008; Redondo and Alvarez-Pellitero, 2009; Kang et al., 2016). By using lectins with distinct sugar affinities – such as the WGA, soybean agglutinin, *Bandeiraea simplicifolia* lectin, concanavalin A (Con-A), *Ulex europaeus* agglutinin and the *Sambucus nigra* lectin – these studies have pointed to a considerable diversity in the carbohydrate composition of myxozoan parasites. At the same time, the binding of WGA and/or Con-A to the valves and polar capsules of myxospores from multiple species, including *Ceratomyxa* spp., *Kudoa* sp., *Myxobolus* spp., *Sphaerospora* spp. and *E. mugilis*, points towards the existence of some degree of structural conservation across species (Lukeš et al., 1993; Muñoz et al., 1999, 2000). Here, we show that WGA binds strongly to *E. mugilis* actinospores, in addition to myxospores (Muñoz et al., 1999), suggesting similarities in the surface carbohydrate composition of both *E. mugilis* transmission stages. The biological implications of this observation, as well as the exact actinospore molecular component(s) that WGA binds to in *E. mugilis*, remain unknown. Due to the high affinity of WGA to *N*-acetyl-d-glucosamine (Nagata and Burger, 1974), the monomeric units of chitin (Gooday, 1990), it has long been proposed that this polymer is an important structural component of myxozoan spores (Lukeš et al., 1993). Although WGA can also bind to sialic acid residues (Monsigny et al., 1980), experiments using Calcofluor White stain (Lukeš et al., 1993), a fluorescent dye with affinity to polysaccharides containing contiguous β-1,4-linked d-glucopyranosyl units such as chitin (Moore, 1990), and succinylated WGA, a lectin that specifically binds to *N*-acetyl-d-glucosamine but not to sialic acid residues (Monsigny et al., 1980), further support the hypothesis that the native WGA is recognizing chitin in the valves and polar capsules of spores (Kaltner et al., 2007; Kang et al., 2016).

Chitin is a highly abundant polysaccharide present in fungal cell walls, arthropod cuticle, crustacean exoskeletons, mollusc shells, as well as in other invertebrates (Muzzarelli, 2011), such as cnidarians (Vandepas et al., 2023). Similar to what happens in other organisms, chitin may have an important structural role in myxozoan parasites, providing structural stability and protection against unfavourable environmental conditions and mechanical stress. Interestingly, in contrast to our findings, a previous study showed that WGA stains the sporoplasm and polar capsules, but not the valve cells of *M. cerebralis* triactinomyxon spores (Kaltner et al., 2007). We may speculate that these differences among species are a consequence of distinct adaptations for actinospore survival. Indeed, whereas the portals of entry of *M. cerebralis* actinospores are the secretory openings of the mucous cells of the epidermis, the gill respiratory epithelium and the buccal cavity (Sarker et al., 2015), *E. mugilis* actinospores most likely enter the fish host through its digestive tract, as suggested for the closely related parasite species *Ellipsomyxa gobii* (Køie et al., 2004). Thus, the presence of chitin in the valves may confer additional resistance to *E. mugilis* actinospores, helping them survive the harsh passage through the digestive tract of the host. This hypothesis is supported by the presence of chitin and other *N*-acetylglucosamine containing components in the cyst wall of several pathogenic protists that also have to survive passage through the host digestive tract, such as *Entamoeba* spp. and *Giardia lamblia* (Steinfeld et al., 2019). Finally, the biological significance of the WGA-binding pattern in *E. mugilis* actinospores is unknown; future experiments will hopefully provide further insights into this matter.

### *Optimizing a FACS protocol for the purification of viable* Ellipsomyxa mugilis *actinospores*

Although the progression of *E. mugilis* infection in polychaetes was continuously monitored to select the individual with the most advanced infection, the collected parasite suspension often contained both mature and immature actinospores. This occurs because the formation of *E. mugilis* actinospores within the host is not a completely synchronous process (Rangel et al., 2009). Immature actinospores with reduced viability outside the host could interfere with subsequent experiments, leading, for example, to the overestimation of the inoculum size in experimental infections or the erroneous interpretation of transcriptomic and proteomic data. To avoid such challenges, we have developed a protocol that allows the isolation of viable *E. mugilis* actinospores using DAPI and PI. Curiously, in our cell viability assay, we observed that non-viable actinospores could not be detected by flow cytometry and microscopy (data not shown) after staining with 7-AAD. Several reasons may explain these observations. Firstly, the degree of membrane permeabilization of immature and heat stressed actinospores may be insufficient to allow the passage of high molecular weight cell membrane-impermeant dyes such as 7-AAD (1270.43 g mol^−1^), but sufficient for lower molecular weight dyes, such as DAPI (350.3 g mol^−1^), PI (668.4 g mol^−1^) or even trypan blue (960.81 g mol^−1^). Alternatively, the characteristically low GC content of myxozoan genomes (Alama-Bermejo and Holzer, 2021) may in part contribute to the 7-AAD weak binding to *E. mugilis* DNA, as this fluorescent dye has affinity to GC-rich sequences (Modest and Sengupta, 1974). Even following fixation and permeabilization with Triton-X, 7-AAD failed to stain actinospore nuclei, in contrast to the nuclei of host cells (data not shown).

Hence, using DAPI and PI in combination with WGA-488, we established a FACS-based purification protocol that consistently yielded *E. mugilis* actinospores with approximately 98% viability, a value similar to that obtained for the purification of *S. molnari* blood stages from whole fish blood using DEAE-C (Born-Torrijos et al., 2022). Importantly, the use of a viability dye enabled the exclusion of most WGA⁺ host cells during sorting, resulting in the removal of approximately 93% of host cells initially collected with the parasites. The remaining 7% showed significantly lower fluorescence than actinospores, which minimized their impact on the sorting process, given that the threshold used for sorting excluded low-intensity events. Shivam and colleagues obtained promising results regarding the enrichment of parasite reads in their RNA-seq data using FACS, revealing the untapped potential of this technique for much-needed Myxozoa multi-omics studies (Shivam et al., 2023). Regardless of the methodology used for the purification of myxozoan parasites, future studies should consider incorporating assays for the absolute quantification of host DNA in the sorted samples while optimizing the purification protocol.

In conclusion, this study successfully achieved sustained production of *E. mugilis* actinospores in an indoor mesocosm system. In addition, we developed a simple, fast and efficient protocol for purifying viable actinospores from the surrounding host material, which is crucial for generating high-quality next-generation sequencing data, among other applications. Our work paves the way for future research focused on studying actinospore infectivity to the fish host.

## Supporting information

Sá et al. supplementary material Sá et al. supplementary material

## Supplementary material

The supplementary material for this article can be found at https://doi.org/10.1017/S0031182025100784.
