# Supplementary material for: Sustained production and purification of Ellipsomyxa mugilis actinospores in a laboratory mesocosm
Source: Parasitology. 2025 Aug 29;152(11):1144–53. doi: 10.1017/S0031182025100784 (PMC12921276; doi:10.1017/S0031182025100784)
Supplement: Sá et al. supplementary material [file S0031182025100784sup001.docx]

**
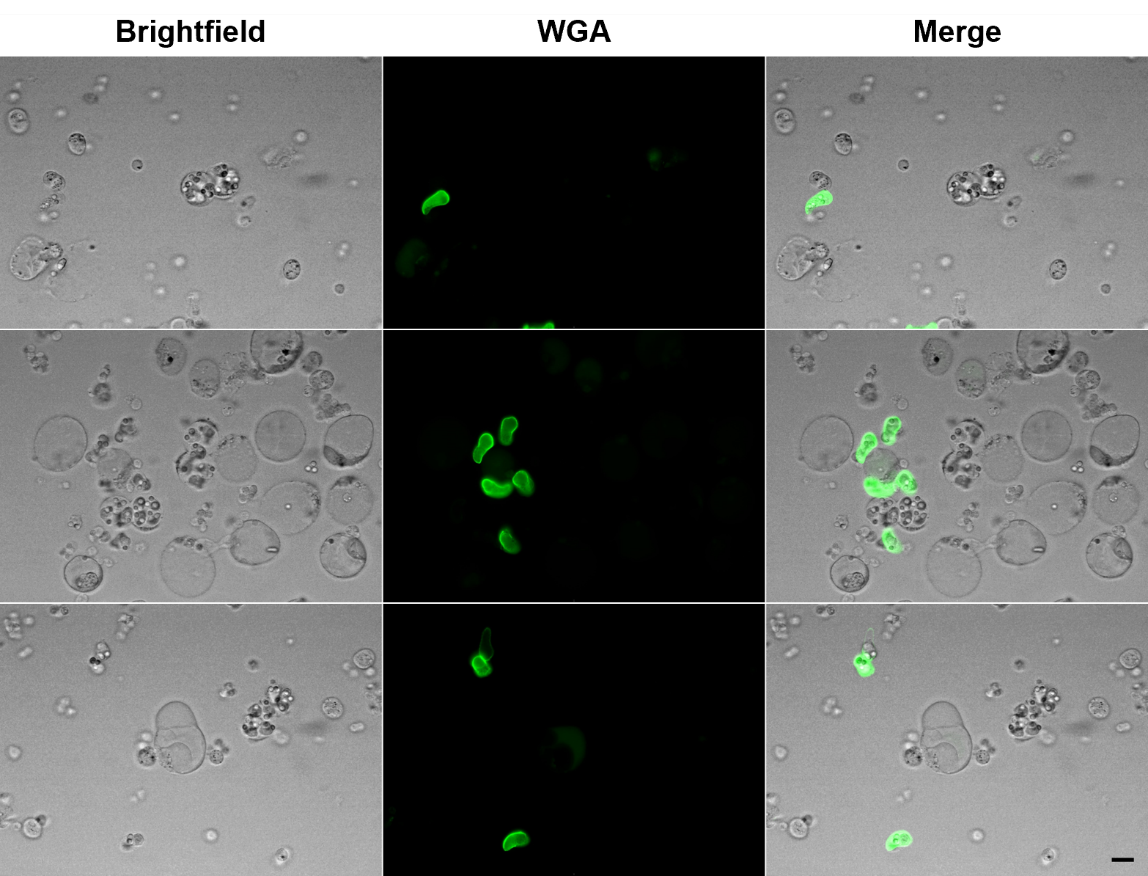
**

**Supplementary figure 1.** Wheat germ agglutinin binds preferentially to *Ellipsomyxa mugilis* actinospores rather than *Hediste diversicolor* coelomic cells. Representative brightfield and immunofluorescence images of *E. mugilis* parasites and host cells stained with Alexa fluor 488-conjugated wheat germ agglutinin (WGA, green) acquired using widefield microscopy. Scale bar, 15 µm.

**
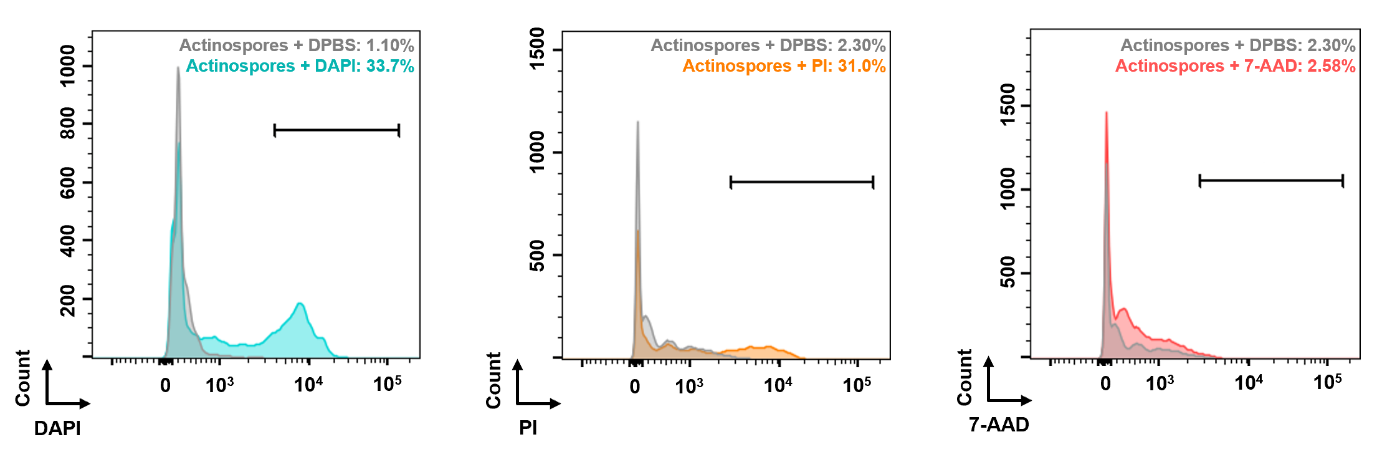
**

**Supplementary figure 2.** Quantitative assessment of *Ellipsomyxa mugilis* actinospore viability by flow cytometry using different nucleic acid-binding dyes. Histograms show the percentage of DAPI^+^ (left panel), propidium iodide^+^ (PI; middle panel), and 7-Aminoactinomycin D^+^ (7-AAD; right panel) actinospores with 31% viability, quantified previously with the trypan blue-exclusion assay. Histograms are representative of two independent experiments using actinospores collected from different polychaetes.

**
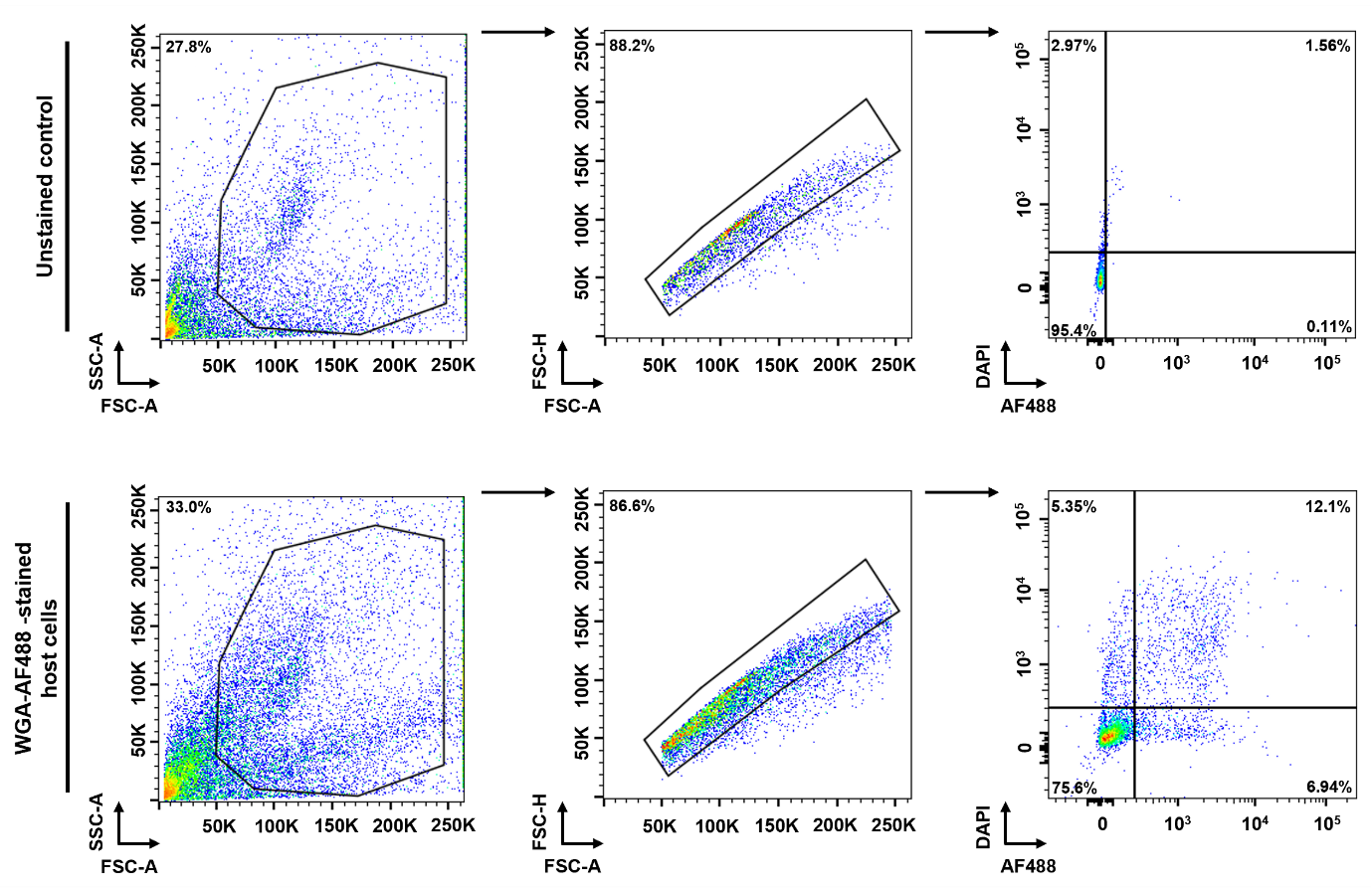
**

**Supplementary figure 3.** Binding of wheat germ agglutinin to *Hediste diversicolor* coelomic cells. Flow cytometry dot plots showing the gating strategy used to identify AF488^+^/DAPI^-^ coelomic cells collected from an uninfected polychaete. From left to right: cells were first gated based on the forward and side scatter properties, then cell doublets were excluded, and finally identified gated based on the AF488 and DAPI fluorescence intensity. As negative control, cells incubated with DPBS were used (upper panel).

**
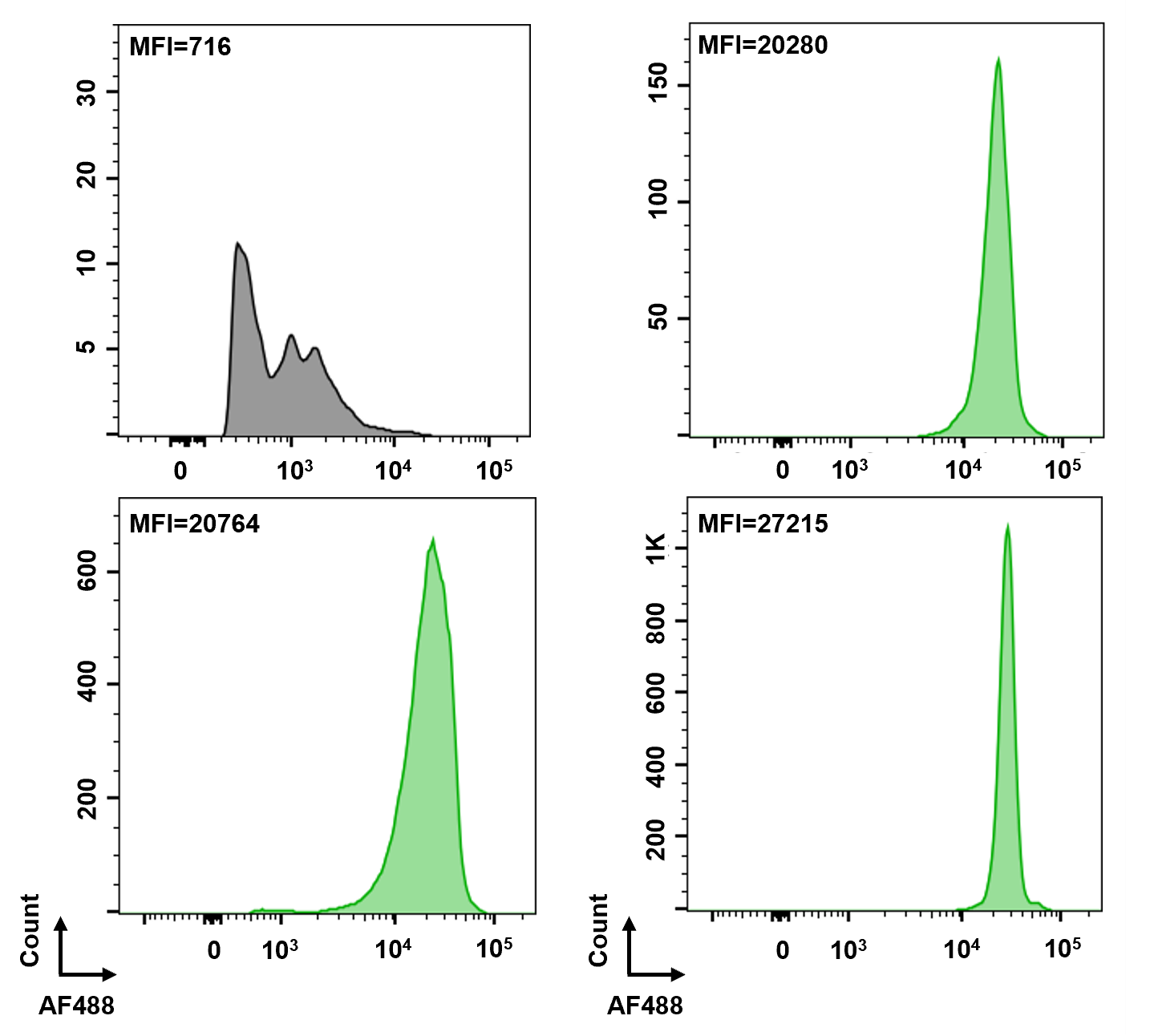
**

**Supplementary figure 4.** Fluorescence intensity of Alexa fluor 488-conjugated wheat germ agglutinin binding to *Hediste diversicolor* coelomic cells and *Ellipsomyxa mugilis* actinospores. Cells were first gated based on the forward and side scatter properties, then gated for single cells, and identified using a AF488^+^/DAPI^-^ gate. Each histogram represents an independent experiment and shows the median fluorescence intensity (MFI) and distribution of the AF488 signal for the AF488^+^/DAPI^-^ host cell (grey) and actinospore (green) populations.
